# Fate of ptaquiloside—A bracken fern toxin—In cattle

**DOI:** 10.1371/journal.pone.0218628

**Published:** 2019-06-21

**Authors:** Paulo César dos Reis Aranha, Lars Holm Rasmussen, Godelind Alma Wolf-Jäckel, Henrik Michael Elvang Jensen, Hans Christian Bruun Hansen, Christian Friis

**Affiliations:** 1 Department of Veterinary and Animal Sciences, University of Copenhagen, Frederiksberg, Denmark; 2 Department of Technology, University College Copenhagen, Copenhagen, Denmark; 3 Department of Plant and Environmental Sciences, University of Copenhagen, Frederiksberg, Denmark; University of Kentuky, UNITED STATES

## Abstract

Ptaquiloside is a natural toxin present in bracken ferns (*Pteridium sp*.). Cattle ingesting bracken may develop bladder tumours and excrete genotoxins in meat and milk. However, the fate of ptaquiloside in cattle and the link between ptaquiloside and cattle carcinogenesis is unresolved. Here, we present the toxicokinetic profile of ptaquiloside in plasma and urine after intravenous administration of ptaquiloside and after oral administration of bracken. Administered intravenously ptaquiloside, revealed a volume of distribution of 1.3 L kg^-1^ with a mean residence-time of 4 hours. A large fraction of ptaquiloside was converted to non-toxic pterosin B in the blood stream. Both ptaquiloside and pterosin B were excreted in urine (up to 41% of the dose). Oral administration of ptaquiloside via bracken extract or dried ferns did not result in observations of ptaquiloside in body fluids, indicating deglycosolidation in the rumen. Pterosin B was detected in both plasma and urine after oral administration. Hence, transport of carcinogenic ptaquiloside metabolites over the rumen membrane is indicated. Pterosin B recovered from urine counted for 7% of the dose given intravenously. Heifers exposed to bracken for 7 days (2 mg ptaquiloside kg^-1^) developed preneoplastic lesions in the urinary bladder most likely caused by genotoxic ptaquiloside metabolites.

## Introduction

**Fig 1:**
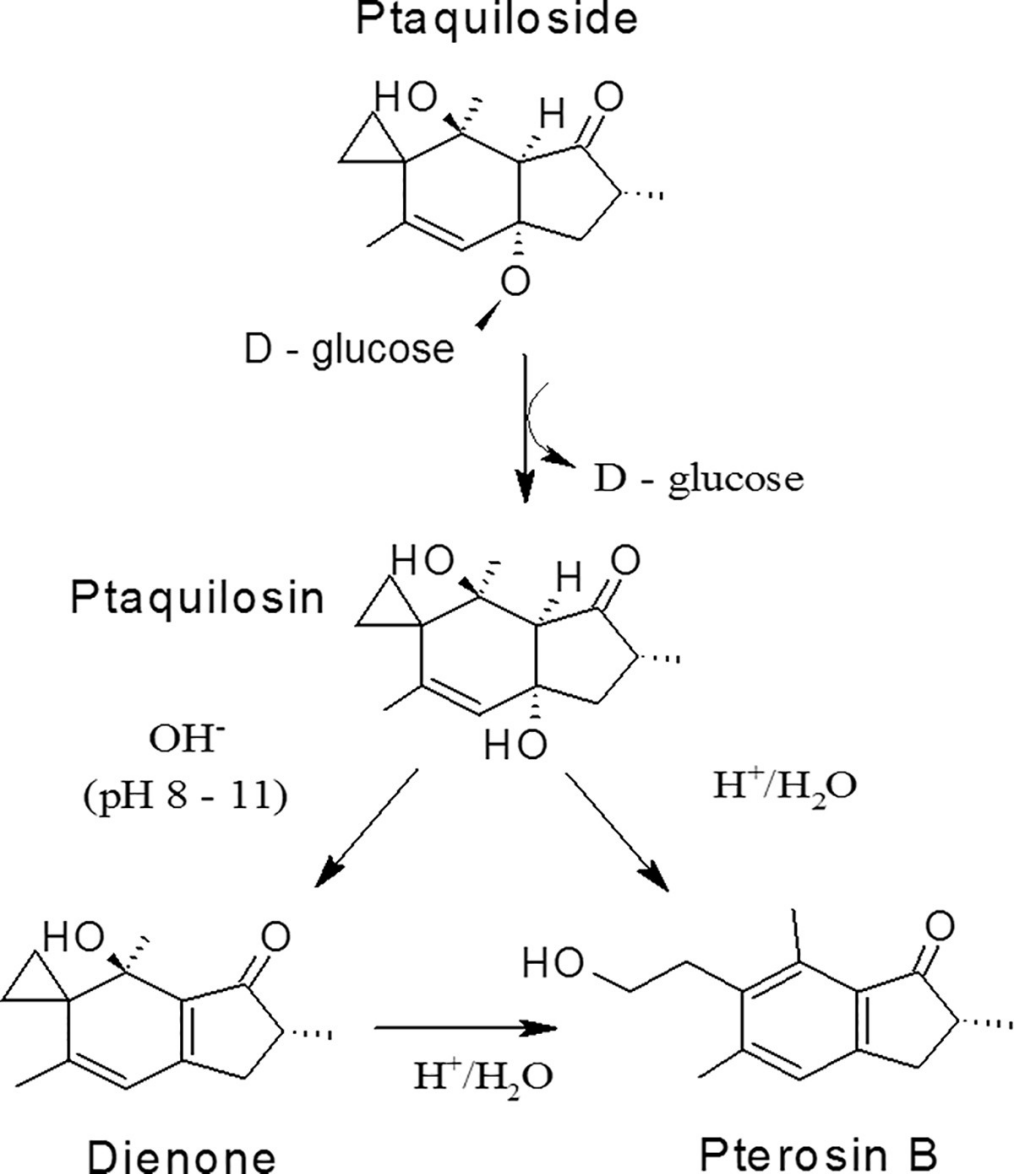
Acid and base catalysed hydrolysis of PTA [14].

Ptaquiloside (PTA) is the most well-studied member of the group of highly toxic and genotoxic illudane glucosides. The group comprises compounds like caudatoside, ptesculentoside, and ptaquiloside Z, all glycosides comprising a reactive cyclopropane ringsystem. PTA is present in many fern species, including the widespread bracken ferns (Fig 1; [1–4]). Ingestion of bracken by ruminants is associated with several diseases, including acute haemorrhagic disease (calves and sheep); bright blindness (sheep); bovine enzootic haematuria (cattle), and upper alimentary carcinoma (cattle) [5–8].

Bracken is the common name used for the 15 different *Pteridium* species of which *P*. *aquilinum* (L.) Kuhn is the most abundant taxa in Europe [1,9]. Brackens have world-wide distribution and are usually found in dense stands inside forests and plantations, at former farmland, at heather, in mountains and as natural part of the bush. The ferns are commonly growing at the edge of paddocks from where they extend belowground rhizomes into the paddocks. Cattle browse on bracken in times of food scarcity, especially in extensive cattle production systems where paddocks are unmanaged [9,10]. PTA is widely distributed among the species and can reach frond concentrations of approx. 40,000 μg/g. In general, the content of PTA is between 500 and 5,000 μg/g. The concentration is variable and generally highest among newly emerged crosiers [2,3,11–13].

Concerns of Human exposure to PTA have increased during the last decade. Humans may be exposed to bracken carcinogens by consumption of meat or milk from cattle grazing on pastures with bracken. Milk from cattle fed bracken has proved mutagenic to *Salmonella typhimurium* TA100 and capable of inducing tumours in rats [15]. Similarly, milk containing PTA induced the acute haemorrhagic syndrome in calves [16]. PTA has been reported in milk from cows fed bracken under experimental conditions [17,18], and in milk from animals grazing bracken [10,19]. In 2011, PTA like-compounds were identified in muscle, liver, kidney and heart tissue from calves fed bracken [20,21]. Therefore, there are strong indications for the potential of bracken toxins to reach people via contaminated milk or meat from cattle in bracken areas. All studies reported above, have used indirect quantification of PTA (derivation into pterosins, e.g. PTB) since direct analysis of PTA in milk was not possible before 2014 [22]. Today, there is still no direct proof of PTA in milk—only evidence of compounds reacting into pterosins under different treatments, e.g. PTA but also the metabolites ptaquilosin and the bracken dienone [22].

PTA is converted to the aglycone ptaquilosin in aqueous solutions upon liberation of glucose and further on to PTB, which is relative stable under laboratory conditions, but rapidly degrades under influence of microorganisms [23]. The molar ratio between PTA and PTB is often smaller than 1 as by-products may form depending on the reaction conditions [3]. The conversion rate is depending on temperature and pH in dilute aqueous solutions. Under alkaline conditions at room temperature (above pH 9), the aglycone ptaquilosin undergoes aromatization resulting in formation of the highly reactive intermediate compound, the so-called bracken dienone [14,24]. The dienone is stable in alkaline solutions at room temperature, but is extremely unstable under acid conditions, being immediately converted to PTB in pure aqueous solutions [14,25]. The dienone acts as an alkylating agent towards nucleophiles (e.g. water, alcohols, amines, amino acids, nucleosides and nucleotides) [14,26]. Thus, bracken fern, PTA, ptaquilosin and the dienone have been related to cytotoxicity [27,28] and genotoxicity [29–31] and immunomodulatory effects [32–34]. Under acidic laboratory conditions, PTA is expected to transform to PTB via ptaquilosin only. However, in complex biological matrices, such as cattle rumen juice (pH approx. 6.7), plasma (pH approx. 7.4), urine (pH approx. 8) and milk (pH approx. 6.8) numerous reactions may occur depending on the type of reactive species present and the interactions caused by hydrolysis, enzymes, temperature, plants/water eaten together with bracken etc. [35]. The complex in-vivo conditions may result in deviations from the ideal reaction scheme PTA to PTB with a molar conversion ratio of 1:1 observed under laboratory conditions.

In order to make a proper risk assessment of milk and meat products from cattle living in bracken infested areas, information on the fate of PTA in the bovine is needed. Currently, no information is available except from the indirect observations of PTA in meat and milk referred to above. Accordingly, the aim of the present work was to outline 1) PTA toxicokinetics in cattle through intravenous administration of purified PTA and oral administration of bracken fern, and 2) toxic effects after a short exposure period to the fern.

## Methods and materials

### Ptaquiloside, *Pteridium aquilinum* (bracken fern) extract and dried bracken fern

Bracken fern pinnae were harvested at Præstø Fed, Denmark (61°158’N, 69°72’E) from young and fully developed ferns at the end of May 2012. The material was dried for 72 hours at 40°C and milled to a particle size <2mm. The content of PTA in the dried fern was determined by extracting 500 mg of milled plant material with 25 mL of Milli-Q water for 60 min. The sample was centrifuged for 15 min at 3000 *g* before purification of 4 mL of bracken extract on a Polyamide 6 dried packed column (500 mg). The eluate was diluted 3 times and PTA determined by LC-MS (see below).

Ptaquiloside (CAS no: 87625-62-5; IUPAC name: (2'R,3a'R,4'S,7a'R)-4'-Hydroxy-2',4',6'-trimethyl-3'-oxo-2',3',3a',4'-tetrahydrospiro[cyclopropane-1,5'-inden]-7a'(1'H)-yl β-D-glucopyranoside) was extracted and purified as described by Aranha et al. [22]. The purity of PTA was determined by quantitative H NMR measuring the proton at C9 of PTA (chemical shift of 5.7 ppm) against an internal standard of 0.5 mM of deuterated trimethylsilyl propanoic acid. The purity was 95%.

Bracken extract used for oral administration was obtained by water extraction of the milled dried plant material; 150 g was extracted with 1.5 L of Milli-Q water for 60 min. Afterwards the extract was passed through cheese cloth and centrifuged for 15 min at 3000 *g* before loading on 2 columns dry packed with 15 g Polyamide 6. The concentration of PTA in the bracken extract was determined by diluting the solution 3 times with Milli-Q water and determination by LC-MS (see below). The extracts were kept at 5°C until use within 48 hours. One mL of the extract was collected immediately before administration to confirm that PTA remained intact during storage.

Dried bracken used in the one-week-exposure study was milled and divided in several portions of 59, 70, 58 and 85 g which correspond to a PTA dose of 2.5 μmoles kg^-1^ of body weight (bw) (1 mg kg^-1^). Molasses was acquired from Danish Agro (Karise, Denmark) and 50 to 100 g was added to each portion of dried fern together with 100 to 150 mL of tap water.

### Chemicals, reagents and solid phase extraction cartridges

Polyamide 6 was obtained from Fluka (Steinheim, Switzerland). *B-Glucuronidase*/*Arylsulfatase* from *Helix pomatia* L. was purchased from Roche (Mannheim, Germany). Oasis HLB SPE cartridges were used for sample preparation (Waters, Hedehusene, Denmark). All other chemicals were obtained from commercial sources and were of analytical grade [22].

### Animals

#### Single dose trials

Two healthy Jersey heifers weighing 92 and 98 kg (5 months old) were obtained from a local dairy farm for single dose studies (no’s 4848 and 4849; Svanholm, Skibby, Denmark). The heifers had never been exposed to PTA or to bracken fern. They were kept in individual boxes during the trial periods with bedding consisting of straw and wood shavings. The heifers were fed with complete feed concentrate (Grønkalv Maxi; DLG Copenhagen, Copenhagen, Denmark) twice daily according to nutrition requirements (1.25–2.00 kg per meal) [36]. Barley straw and water were provided *ad libitum*.

#### One week repeated dose trial

The two heifers used for the single dose trials were also used for the one week repeated dose study together with an additional two Jersey heifers (no’s 1550 and 1567; Harald Hansen Eftf. I/S, Slangerup, Denmark) weighing 163 to 237 kg. The additional two heifers had never been exposed to PTA or to bracken fern. A wash-out period of 20 days between the last single-dose experiment and the one week repeated dose trial was established for heifers no’s 4848 and 4849.

### Experimental protocol, in vivo studies

#### Single dose studies

Two heifers (no’s 4848 and 4849) were used in a series of five experiments (Table 1) in a cross-over design including intravenous administration of pure PTA, oral administration of bracken extract and oral administration of dried bracken fern. At least 2 weeks elapsed between each experiment.

**Table 1:** Overview of PTA doses used in the five animal experiments.

Experiment number		1		2		3		4		5	
		Intravenous		Oral extract		Oral fern		Intravenous		Oral extract	
**Heifer no**		4848	4849	4848	4849	4848	4849	4848	4849	4848	4849
**PTA dose**	mg kg^-1^	0.21		0.41		1.6		0.092	0.056	2	
	μmoles kg^-1^	0.53		1.03		4.02		0.231	0.141	5.02	
**Weight**	kg	98	92	108	102	118	112	144	143	159	149
**Age**	months	5.5		6		6.5		8		8.5	

Intravenous administration of PTA. In Experiment 1 the dose of purified PTA (0.21 mg kg^-1^ body weight (b.w.) / 0.527 μmoles kg^-1^) was dissolved in 10 mL of sterile water in a brown glass vial 1 hour before administration and protected from light. The solution was injected through a catheter (Cavafix Certo: 45 cm, 16 G; B. Braun Melsungen AG, Melsungen, Germany) placed in the right jugular vein. Blood samples were collected through another catheter placed in the left jugular vein. Blood samples were collected in heparinized glass tubes before and at predetermined times (10, 20, 30, 45 min and 1, 2, 4, 6, 8, 12, 21 and 24 hours) after PTA administration. The blood was centrifuged for 15 min at 3000 *g* to separate plasma which then was stored immediately at -18°C until assayed within a week following the method of Aranha *et al*. [22]. Urine was collected quantitatively in urinary bags (Ratiomed GmBH, München, Germany) through a Foley catheter (Rusch Silasil no 16; Teleflex, Milan, Italy) inserted into the bladder. Samples were collected before and at predetermined times (1, 2, 4, 6, 8, 12, 20 and 24 hours) after PTA administration. Two replicates from each sample were diluted 10 times (0.5 mL: 4.5 mL) with 0.1 M acetic acid, buffered to pH 5 with ammonium acetate and frozen immediately at -18°C until assayed within a week following the method of Aranha *et al*. [22]. In the second intravenous experiment (Experiment 4) the PTA was administered intravenously as in Experiment 1, although the dose of PTA was lower, 0.092 and 0.056 mg kg^-1^ b.w (0.23 and 0.14 μmoles kg^-1^. for heifer no 4848 and no 4849, respectively. In this experiment, PTA was dissolved in sterile water immediately before injection.

Oral exposure, bracken extract. Bracken extract was administered orally by gavage in Experiment 2 (170 and 172 mL) and Experiment 5 (820 and 850 mL), to obtain PTA doses of 0.41 and 2 mg kg^-1^ b.w (1.025 and 5.000 μmoles kg^-1^), respectively. Blood and urine samples were collected up to 48 hours after gavage and samples were processed and stored as described previously.

Oral exposure, dried bracken fern. In Experiment 3, dried bracken fern was milled to a fine powder. Immediately before administration, the powder was mixed with water to produce a slurry (15 g of bracken per 100 mL of water). The slurry was administered orally by gavage (400 mL and 380 mL) corresponding to a PTA dose of 1.6 mg kg^-1^ b.w (4 μmoles kg^-1^). Blood and urine samples were collected up to 72 hours after gavage and the samples were processed and stored as described above.

#### One-week repeated dose trial

**Fig 2:**
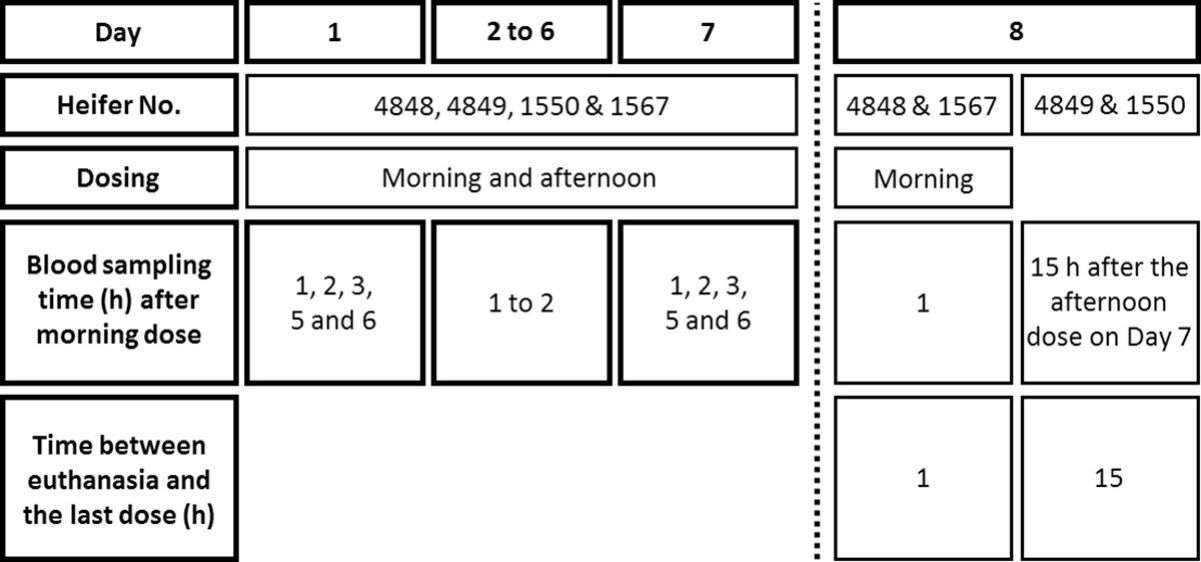
Overview of the exposure study protocol.

All four heifers were fed dried bracken powder (top-dressed and molasses added). twice a day during a period of 7 days providing a daily PTA dose of 2 mg kg^-1^ (5 μmoles kg^-1^). On Day 8, the heifers no 4848 and no 1567 were additionally fed in the morning and euthanized 1 hour after using a captive bolt pistol and exsanguination. The two other heifers (no 4849 and no 1550) were euthanized 15 h after the last dose on Day 7 (Fig 2).

Blank blood samples were collected before the first dose was given. On Day 1 and Day 7 blood samples were collected after the morning dose at predetermined time points between 1 and 6 h after dosing (1, 2, 3, 5 and 6 hours). On Day 2 to Day 6 blood samples were collected daily 1 hour after 50% or more of the morning feed had been ingested, since the bracken fern was not eaten instantly. On Day 8 blood samples were collected just before euthanasia. Blood samples were processed and stored as described above.

### Pathology

Necropsy was performed within 1.5 hours after euthanasia. Following gross examination, tissue samples were collected for histological examination from the following organs: Sternal bone marrow, heart, rumen, omasum, abomasum, small intestine, liver, kidney, and urinary bladder. The samples were fixed in 10% neutral buffered formalin for five to seven days, processed through graded concentrations of ethanol and xylene and embedded in paraffin. Sections were cut at 2 to 3 μm for liver, heart, rumen, omasum, abomasum, small intestines, kidney and urinary bladder and at 3 to 5 μm for bone marrow. Thereafter, sections were stained with haematoxylin and eosin.

### Incubation of bracken extract in rumen liquid, in vitro experiments

Samples of rumen contents were collected from two rumen fistulated Jersey cows. The cows were fed daily with 6 kg of complete feedingstuff (Danish Ragna Grønmix; Danish Agro, Karise, Denmark) and 2 kg of hay. Barley straw and water were provided *ad libitum*. The rumen liquid fraction was obtained by passing the digest through a 250 μm nylon filter. From each cow, 150 mL rumen liquid was pre-incubated at 37°C under CO_2_ atmosphere for 10 min before 5 mL of bracken extract was added in order to obtain an initial PTA concentration of 17 μM. PTA stability was assessed by removal of 2.5 mL sample for PTA determination after incubation for 0, 5, 15, 30, 60 and 120 min at 37 °C. The sample was immediately centrifuged for 3 min at 8,500 *g* and analysed following the method of Aranha *et al*. [22]. The pH in rumen liquid was determined at the beginning and termination of the experiment.

### Toxicokinetic analysis

Concentrations of PTA in plasma of individual animals were evaluated by non-compartmental analysis. Toxicokinetic variables were calculated using standard kinetic equations. The area under the curve (AUC) and the area under the first moment curve (AUMC) were determined using combined linear and natural logarithm trapezoidal rules. The terminal elimination rate was determined using the last three data points of the curve [37].

The effect of PTA exposure to plasma aspartate aminotransferase (AST), plasma glutamate dehydrogenase (GD), and plasma ornithine carbamoyltransferase (OCT) activity was evaluated using two-way ordinary analysis of variance (ANOVA) using exposure time and animal as variables (PRISM 6.0; GraphPad Software; San Diego, California, USA). A P value <0.05 was considered significant and indicative for changes in activity.

### Analytical conditions and procedures

PTA and PTB were analysed by LC-MS after purification using solid phase extraction (SPE) columns as reported in Aranha *et al*. [22]. The limit of quantification of PTA and PTB in plasma was 3.0 x 10^−3^ μM and 9.30 x 10^−3^ μM, respectively. Limit of quantifications in diluted urine was 13 x 10^−3^ μM for PTA and 8.28 x 10^−3^μM for PTB.

The presence of glucuronides and sulphates was assessed by incubating 1 ml of buffered urine samples in 0.1 M acetic acid buffer (pH 5) with or without addition of 100 μl of *B-*glucuronidase/arylsulfatase at 37°C for 3 hours on a shaking water bath. Afterwards, an aliquot of 950 μl of each samples were cleaned on the Oasis HLB cartridges and analysed as described for urine samples [22].

Rumen liquid samples were cleaned on HLB cartridges and processed as plasma samples. PTA and PTB calibration curves were prepared in ruminal fluid deactivated by boiling for 5 minutes. The recovery of PTA and PTB in ruminal fluid was 74% (± 0.5%) and 80% (± 1.2%), respectively.

Hepatotoxicity was evaluated by determining the plasma levels of AST (37°C), GD (25/26°C) and OCT (37°C) using the Sigma-AST Activity Assay Kit (MAK055-1KT), and the Sigma-Aldrich GD Ammonia assay kit (AA0100), and the method of Tsuchiya et al. [38], respectively.

### Ethics statement

This study was carried out in accordance with the rules and regulations for animal experiments at the University of Copenhagen according to the requisites established by the Danish Acta of Animal Experiments and Acta of Veterinarian. At University of Copenhagen, Department of Experimental Medicine is responsible for the local Animal Care and Use Programme. The study was licensed by the Danish Animal Experiments Inspectorate (permit ID: 2007/561−1434). All possible efforts were done to minimize suffering of the experimental animals. All animals were euthanized by the end of the experiments using a captive bolt pistol and exsanguination.

## Results

### Animal studies

#### Single dose studies

**Fig 3:**
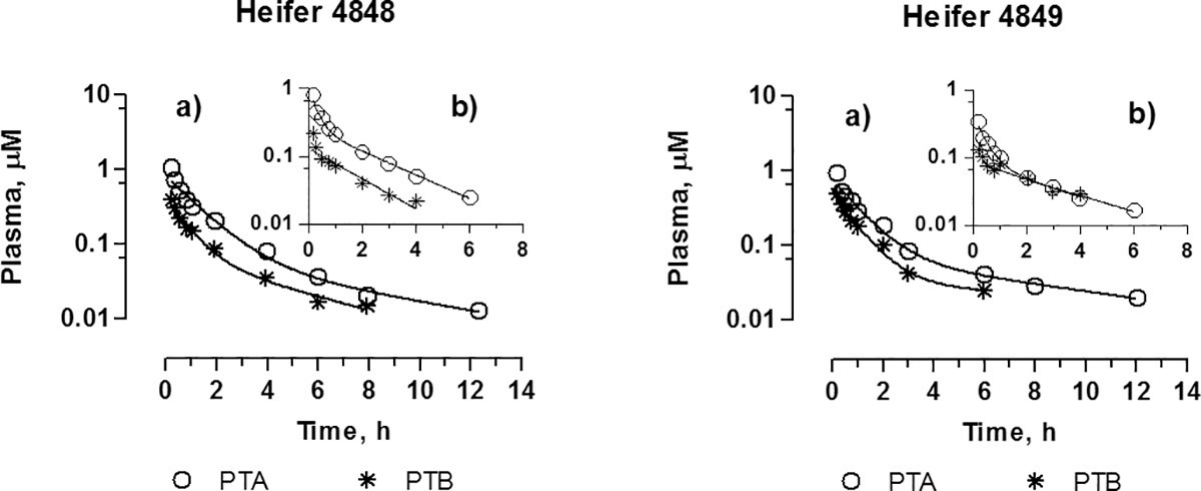
PTA and PTB plasma concentrations over time after intravenous administration of PTA at a dose of: a) 0.527 μmoles kg^-1^ b.w. for both heifers (Experiment 1); b): 0.231 μmoles kg^-1^ b.w. to heifer no 4848 and 0.141 μmoles kg^-1^ b.w. to heifer 4849 (Experiment 4).

Intravenous experiments. The plasma concentration of PTA after intravenous administration of 0.21 mg kg^-1^ b.w. (0.527 μmoles kg^-1^) is shown for each heifer in Fig 3A. The concentration of PTA decreased rapidly and was below the limit of quantification after 12 hours. The average of the mean residence time (MRT) and clearance was 3.4 h and 0.39 L h^-1^ kg^-1^ (Table 2). The apparent volume of distribution at steady state of PTA (Vss) was 1.3 L kg^-1^ indicating an extensive distribution of PTA to tissues.

**Table 2:** Toxicokinetic parameters of PTA and PTB after intravenous injection of PTA.

			Experiment 1		Experiment 2	
	Heifer no		4848	4849	4848	4849
	Dose	μmoles kg^-1^	0.527	0.527	0.231	0.141
**PTA**	**λz**	h^-1^	0.153	0.115	0.379	0.242
	**AUC**_**0-**_∞	μmoles L^-1^ h	1.39	1.35	0.829	0.42
	**MRT**	h	2.99	3.84	2.04	2.48
	**Cl (body)**	L^-1^ h^-1^ kg^-1^	0.38	0.389	0.283	0.334
	**Vss**	L kg^-1^	1.14	1.49	0.567	0.830
**PTB**	**AUC**_**0-**_**∞**	μmoles L^-1^ h	0.596	0.654	0.284	0.319
	**AUC**_**0-**_**∞ PTB / AUC**_**0-**_**∞ PTA**		0.429	0.484	0.343	0.760

**λz** terminal slope; **AUC**_**0-**_∞ area under the curve from 0 to infinitive; **MRT** mean residence time; **Cl (body)** body clearance; **Vss** apparent volume of distribution at steady-state.

Unexpectedly, the PTA metabolite, PTB, peaked at the first sampling point 10 min after administration of PTA. The concentration of PTB declined with time approximately at the same rate as PTA (Fig 3A). Since the aqueous injection solution was prepared approximately 1 hour before administration, it was speculated if a fraction of PTA might have converted to PTB before injection. Consequently, two additional intravenous experiments were conducted although with lower doses of PTA. In these experiments. Purified PTA was dissolved in sterile water immediately before administration in order to ensure that PTA was not hydrolysed to PTB before injection. As shown in Fig 3B similar concentration profiles for PTA and PTB occurred in these experiments compared to the initial ones (Experiments 1 and 4). It is noted that the ratio of AUC _PTB_ to AUC _PTA_ remained at approx. 0.4 for heifer no 4848, but increased from 0.34 to 0.76 for heifer no 4849. This verifies that PTB was not formed in the PTA solution for injection, but that conversion takes place in the animal either by hydrolysis in the blood or by liver metabolism.

**Fig 4:**
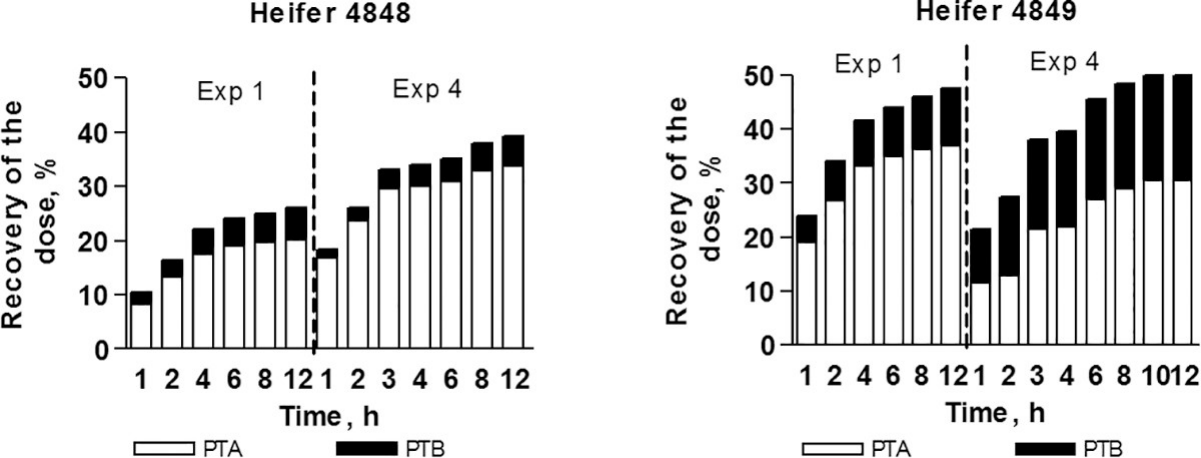
PTA, PTB and total (PTA + PTB) accumulative recovery in urine after intravenous administration of PTA at different dose levels: Heifer no 4848 0.527 dosed with 0.231 μmoles kg^-1^ (Experiment 1 and 4); heifer no 4849 0.527 dosed with 0.141 μmoles kg^-1^ (Experiment 1 and 4).

Both PTA and PTB were detected in urine (Fig 4). Twelve hours after administration 30% of the dose was recovered as PTA and 12% as PTB with the majority excreted during the first 4 hours. The majority of the dose was excreted during the first 4 hours. PTA and PTB were not conjugated with either glucuronic acid or sulphate.

**Fig 5:**
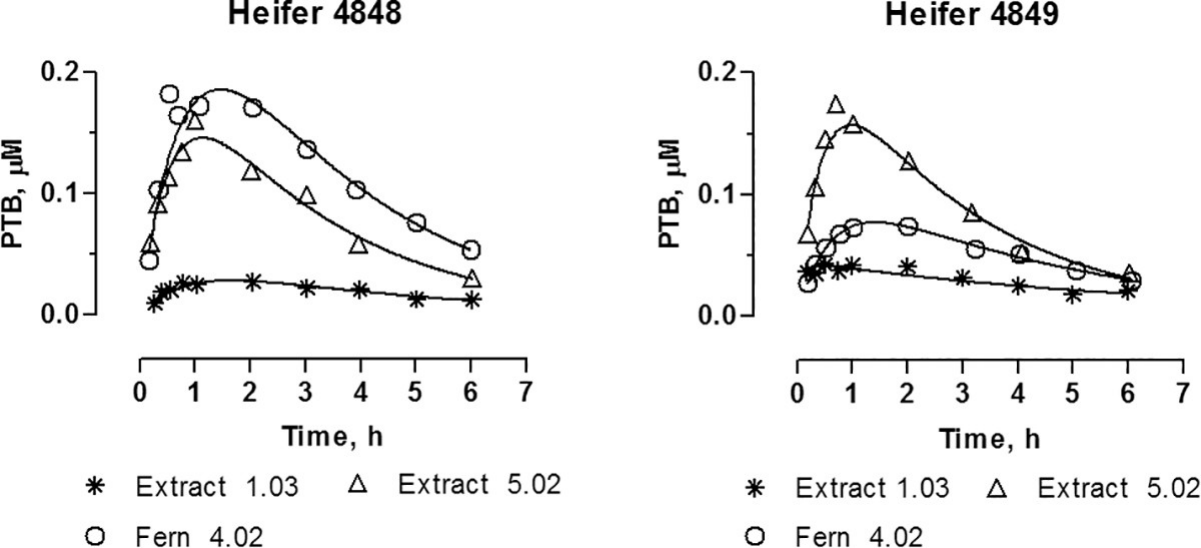
Plasma concentrations over time for PTB after oral administration of PTA as bracken extract at doses 1.03 and 5.02 μmoles kg^-1^ (Extract 1.03 and Extract 5.02), respectively, and as dried bracken fern at dose of 4.02 μmoles kg^-1^ (Fern 4.02).

**Fig 6:**
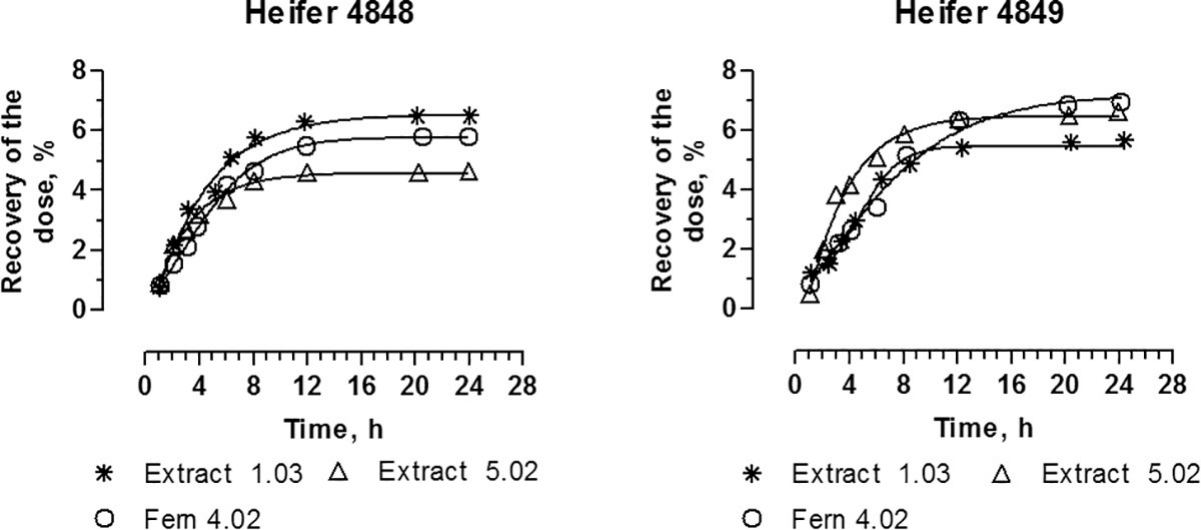
Accumulative recovery of the PTB dose excreted in urine after oral administration of PTA as bracken extract at doses 1.03 and 5.02 μmoles kg^-1^ (Extract 1.03 and 5.02), respectively, and as dried bracken fern with a dose of 4.02 μmoles kg^-1^ (Fern 4.02).

Oral bracken extract and dried fern experiments. PTA was not detected in plasma when the toxin was administered orally either as bracken extract or as dried bracken fern (Experiments 2, 3 and 5). However, PTB was detected in the plasma from the first sample from 10 min after oral administration until 6 or 12 hours after administration (Figs 5 and 6). For both bracken extract and bracken fern the time to maximum concentration in plasma (T_max_) was achieved after approximately 1 hour, the maximum concentration (C_max_) as well as AUC are presented in Table 3. C_max_ increased proportionally with increasing dose of PTA in the bracken extract, while AUC was not linearly correlated with dose. Only PTB was recovered in urine, accounting for up to 7% of the dose within 24 hours (Fig 6).

**Table 3:** Toxicokinetics parameters for PTB in plasma after oral administration of different doses of PTA.

		Experiment 3		Experiment 4		Experiment 5	
		Bracken extract		Bracken extract		Dried bracken	
Heifer no		4848	4849	4848	4849	4848	4849
PTA dose	μmoles kg^-1^	1.03		5.02		4.02	
**λz**	h^-1^	0.234	0.100	0.382	0.290	0.308	0.28
**AUC**_**0-**_**∞**	μmoles L^-1^ h	0.177	0.367	0.594	0.657	0.897	0.428
**MTT**	h	3.82	4.55	2.89	3.02	3.29	3.58
**T**_**max**_	h	2.0	0.5	1.0	0.75	0.5	2.0
**C**_**max**_	μmoles L^-1^	0.028	0.043	0.160	0.174	0.182	0.074

**λz** terminal slope; **AUC**_**0-**_**∞** area under the curve from 0 to infinitive; **MTT** mean transit time; **T**_**max**_ time at maximum concentration; **C**_**max**_ maximum concentration; **F** bioavailability, was calculated by dividing the ratio of **AUC**_**0-**_**∞ oral** by the **dose** and by ratio total PTA **AUC**_**0-**_**∞ IV** by respective **dose.**

#### Incubation of bracken extract in rumen liquid

**Fig 7:**
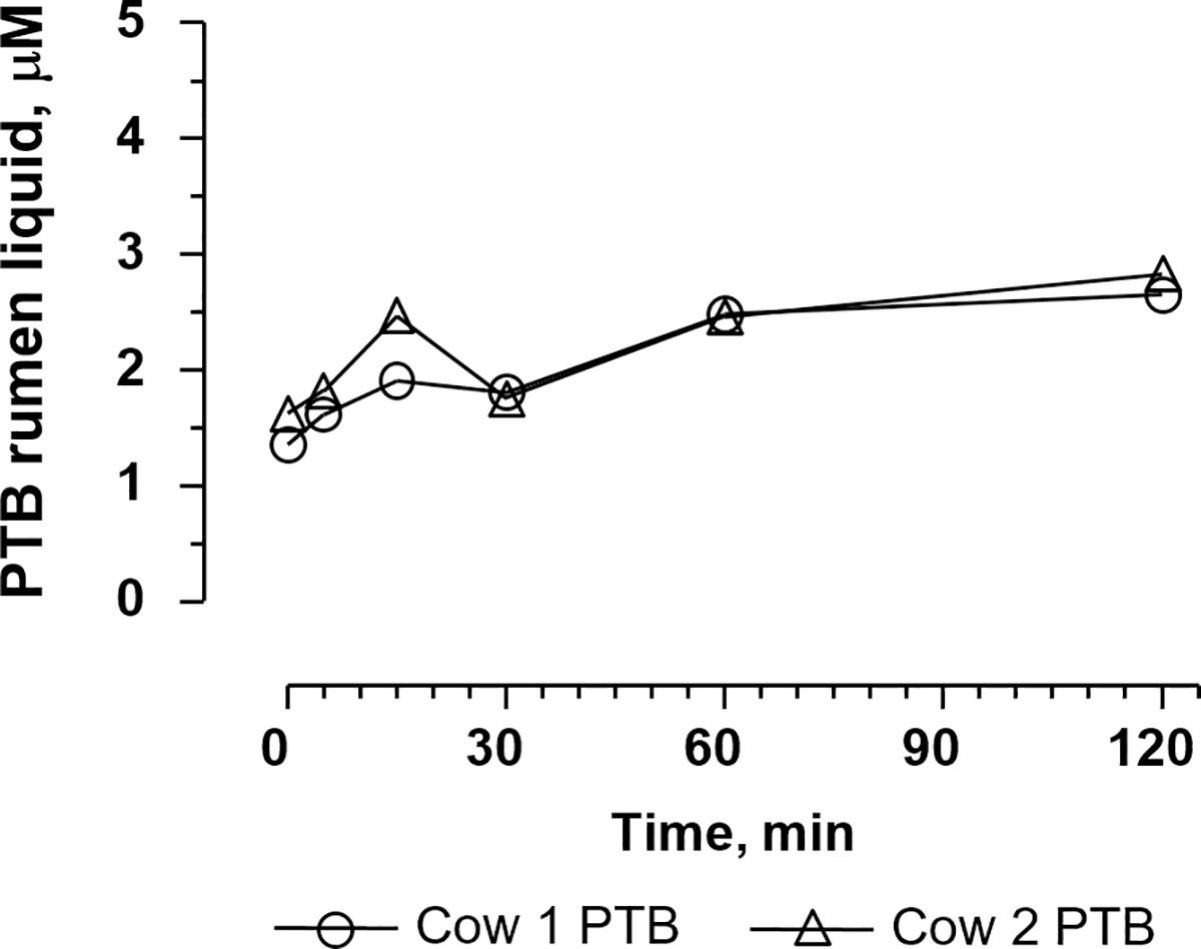
Formation of PTB from PTA in rumen liquid (17 μM) during 120 min at 37 °C. PTA was observed up to approx. 5 min after the experiment began. However, it was not possible to determine the exact concentration due to fast degradation.

PTA concentration decreased during incubation with fresh rumen fluid and was detectable in rumen fluid from cow no 1 only immediately after start, whereas in rumen fluid from cow no 2 PTA could be determined both at the start and after 5 min (Fig 7). The PTB concentration was 1.35 and 1.62 μM at starting time and increased steadily during the first 15 min of incubation (Fig 7) reaching 2.7 μM after 120 min. The pH remained constant (6.6) during the incubation. Adding PTA to boiled or pre-frozen rumen fluid did not reveal any degradation of PTA indicating the importance of activity of microorganisms or enzymes.

#### One week repeated dose trial

**Fig 8:**
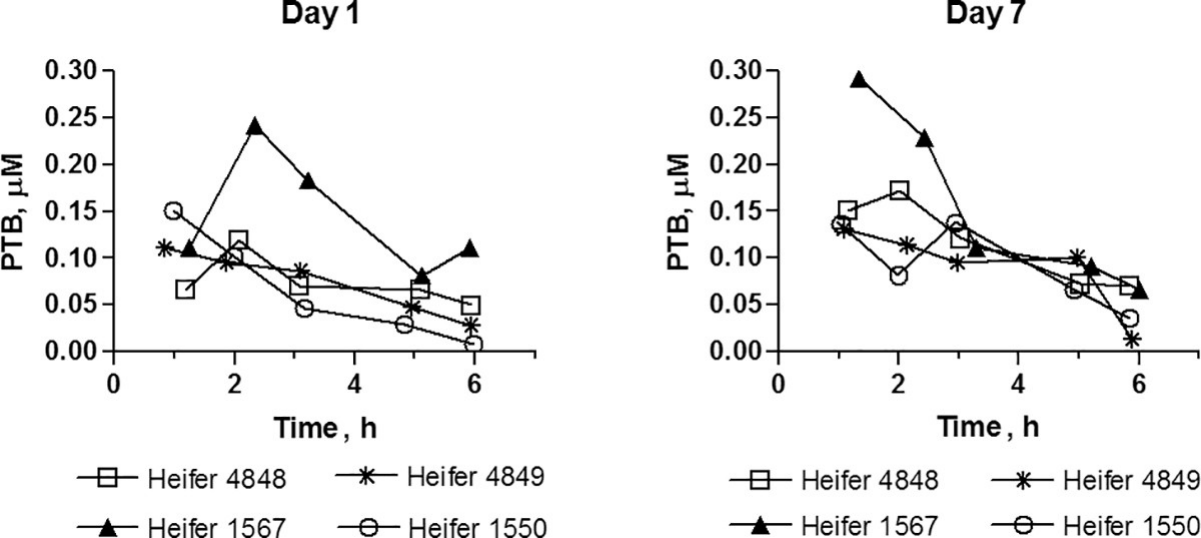
One-week repeated dose trial. PTB plasma profile at Day 1 and Day 7.

**Fig 9:**
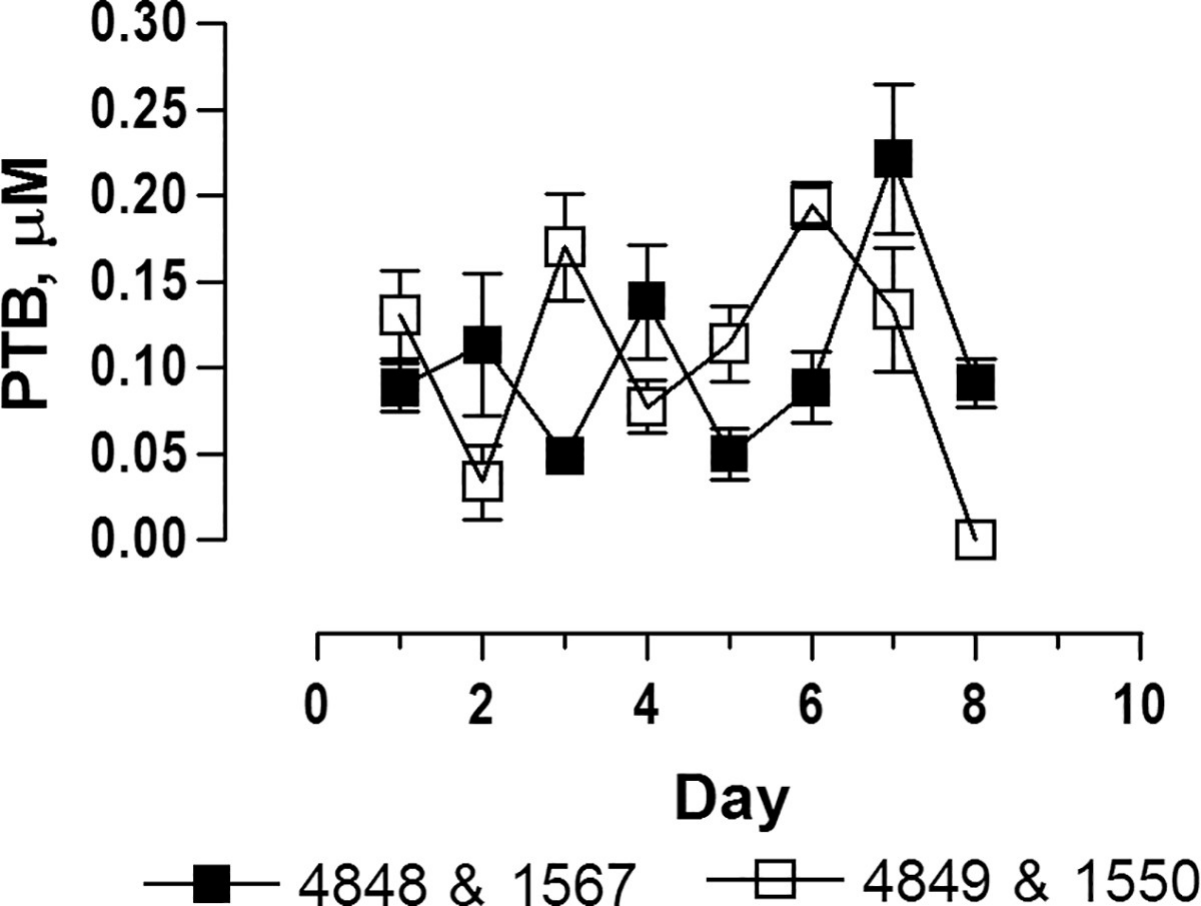
Average of PTB plasma concentrations 1 h after more than 50% of the morning dose has been ingested. Heifers were fed twice a day for 7 days. On Day 8, heifers no 4848 and 1567 received an additional dose one hour before sample collection. Plasma samples from heifer no 4849 and no 1550 was collected 15 h after the afternoon dose at Day 7.

As in single dose studies, PTA was not detected in plasma after oral administration (Fig 8). PTB was detected in the first plasma sample (1 hour after dosing) on Day 1 and Day 7, where after it declined rapidly approaching the limit of detection at 6 hours (Fig 8). The area under curve from 0 to infinity (AUC _0_ -∞) and the mean transit time (MTT) for PTB at Day 1 and Day 7 are shown in Table 4. The mean AUC _0_ -∞ was equal at Day 1 and Day 7 showing that PTB was not accumulated in the organism, although heifer 1550 revealed somewhat lower AUC at Day 1 than at Day 7, which is likely related with slow ingestion of the morning dose at the first day. The average of PTB concentration in plasma was similar between animals during the trial period as shown in Fig 9.

**Table 4:** Area under the curve from 0 to infinitive (AUC_0-_∞) and mean transit time (MTT) on Day 1 and Day 7 on the exposure study.

	Day 1		Day 7	
	AUC_0-_∞	MTT	AUC_0-_∞	MTT
Animal	μmoles L^-1^ h	h	μmoles L^-1^ h	h
**4848**	0.873	4.6	0.968	3.9
**1567**	1.248	4.4	1.264	3.6
**4849**	0.591	3.9	0.558	3.0
**1550**	0.386	2.3	0.606	3.2
**Average**	0.774	3.8	0.849	3.4
**SD**	0.373	1.0	0.332	0.4

Clinical examination and liver enzyme activity. Overall, none of the heifers showed clinical signs of acute or chronic bracken intoxication during the course of experiments. The heifers did not reveal any signs of illness and the refusal of the morning dose on the second day by heifer no 1550 might be related to the low palatability of the fern despite the fact that molasses had been added. No refusal was observed after mixing the bracken doses with complete feeding stuff.

**Fig 10:**
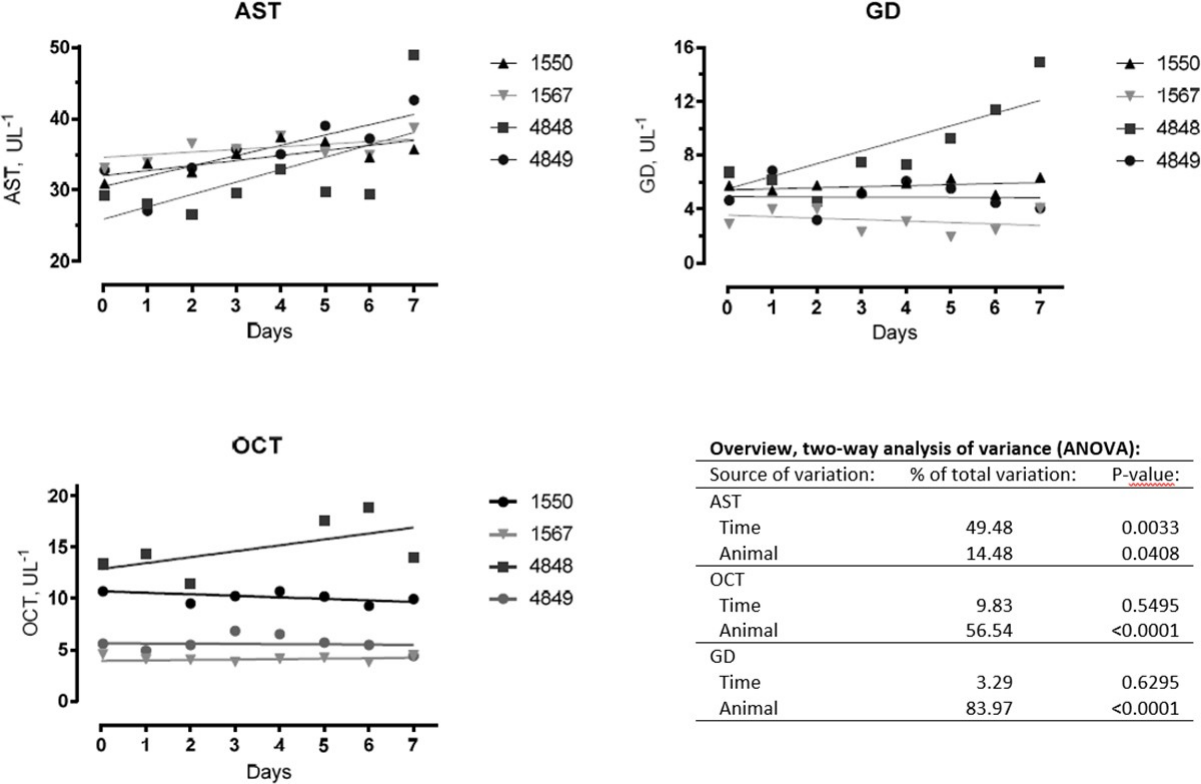
Enzyme activity in plasma before (Day 0) and during the bracken exposure period (Day 1 to Day 8) corresponding to a daily PTA dose of 2 mg kg^-1^. AST, aspartate aminotransferase; GD, glutamate dehydrogenase; OCT, ornithine carbamoyltransferase (OCT). Enzyme activity trend-lines are inserted for individual animals.

In general, significant differences in enzyme activity was observed between animals for AST, GD and OCT (two-way ANOVA P-values: 0.0408, <0.0001 and <0.0001 respectively; Fig 10). A significant increase in AST activity was observed during the course of the experiment (P-value 0.0033) while no effects were observed for GD and OCT activities (P-values: 0.5495 and 0.6295; Fig 10). However, an increase in GD activity was observed for a single animal but with no significant effect on the general model.

### Gross pathology

**Fig 11:**
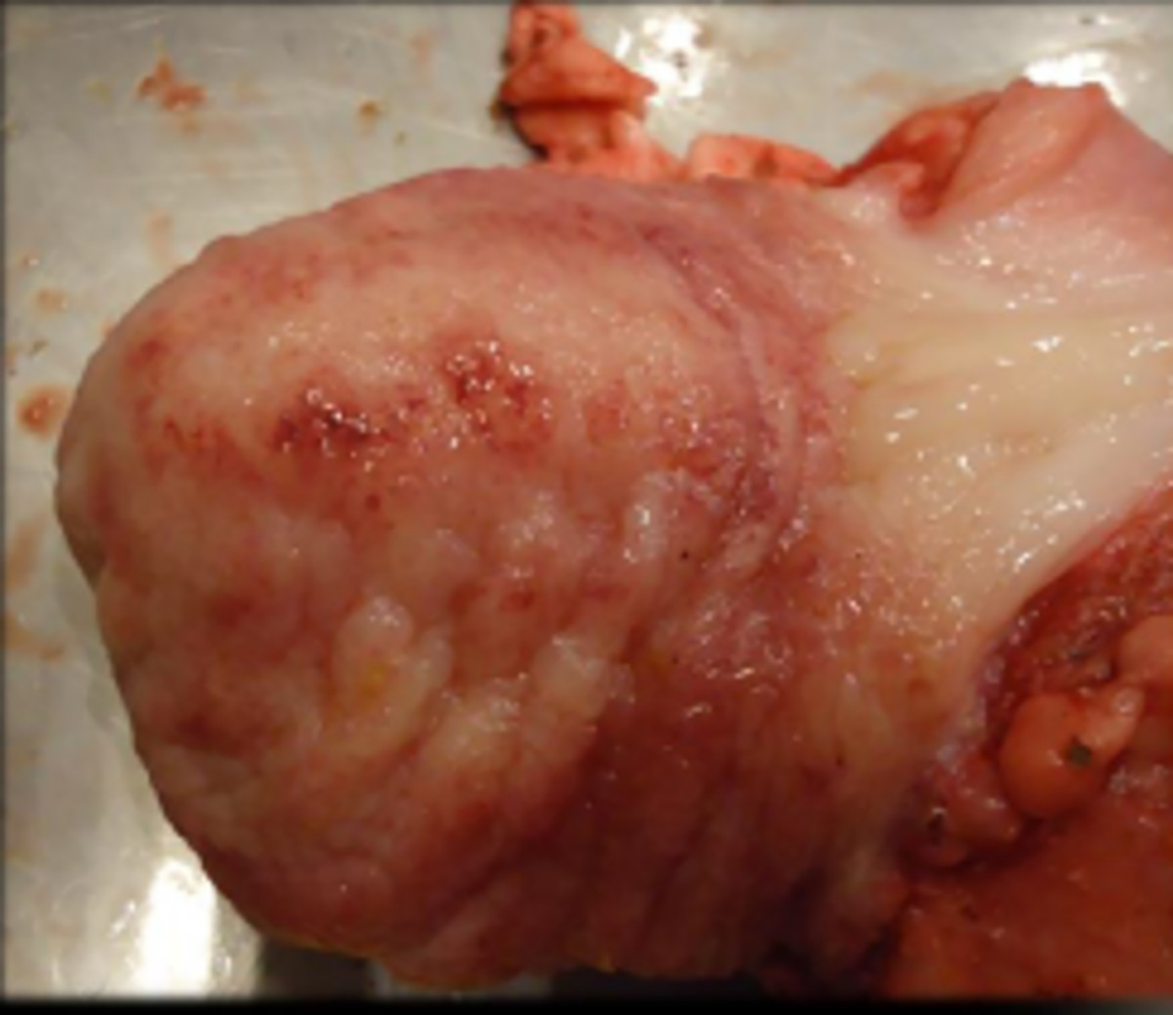
Mucosal surface of the urinary bladder of heifer no 4848. Seven days of bracken fern ingestion (PTA daily dose of 5 μmoles kg-1). Hyperaemia and focal haemorrhages were the most prominent findings.

At gross examination, the following lesions were found in the urinary bladder mucosa: oedema (heifers 1567, 4849, and 1550), focal hyperaemia (heifers 1567 and 4848), and focal small haemorrhages (heifers 4849 and 1550) (Fig 11). Additionally, heifer 4848 was found to be a freemartin and the liver of heifer 1567 was swollen and contained multiple white foci with a diameter of up to 1 mm. No lesions were found in the remaining organs.

#### Histopathology

**Fig 12:**
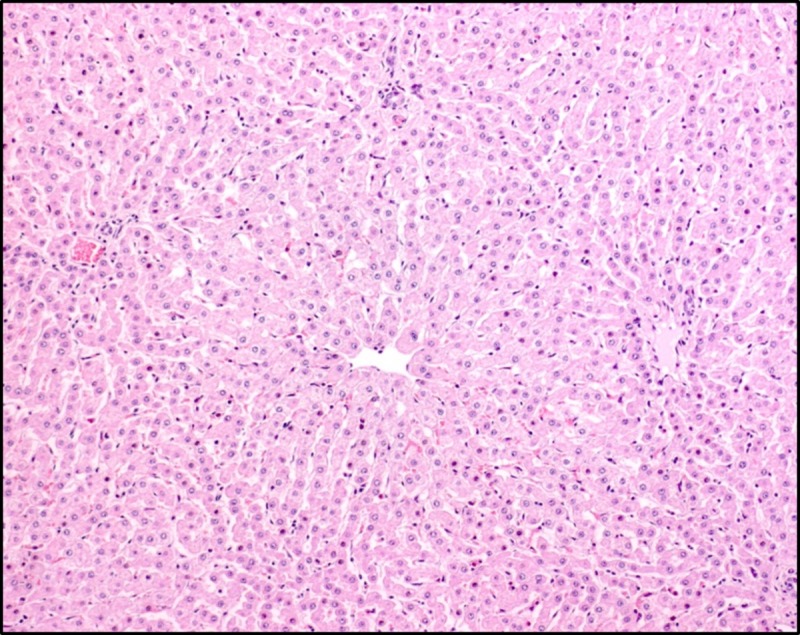
Liver from heifer no 4849. The centrolobular hepatocytes are hypertrophic, which is reflected by their increased size and the finely granular appearance of the cytoplasm. Haematoxylin and eosin.

In all four heifers, histological examination of liver samples revealed moderate centrolobular hypertrophy of hepatocytes with finely granular cytoplasm, as well as biliary and capillary hyperplasia mainly located in the portal areas (Fig 12). Additional findings in the livers were multifocal pericholangic infiltration with mononuclear cells and neutrophils (heifers 1567, 4848, and 1550) as well as multifocal portal fibrosis (heifers 1567, 4849, and 1550) and multifocal diminutive accumulations of mononuclear cells and single neutrophils (heifers 1567, 4849, and 1550). The macroscopic lesions in the liver of heifer 1567 were confirmed histologically to be acute, multifocal micro-abscesses.

**Fig 13:**
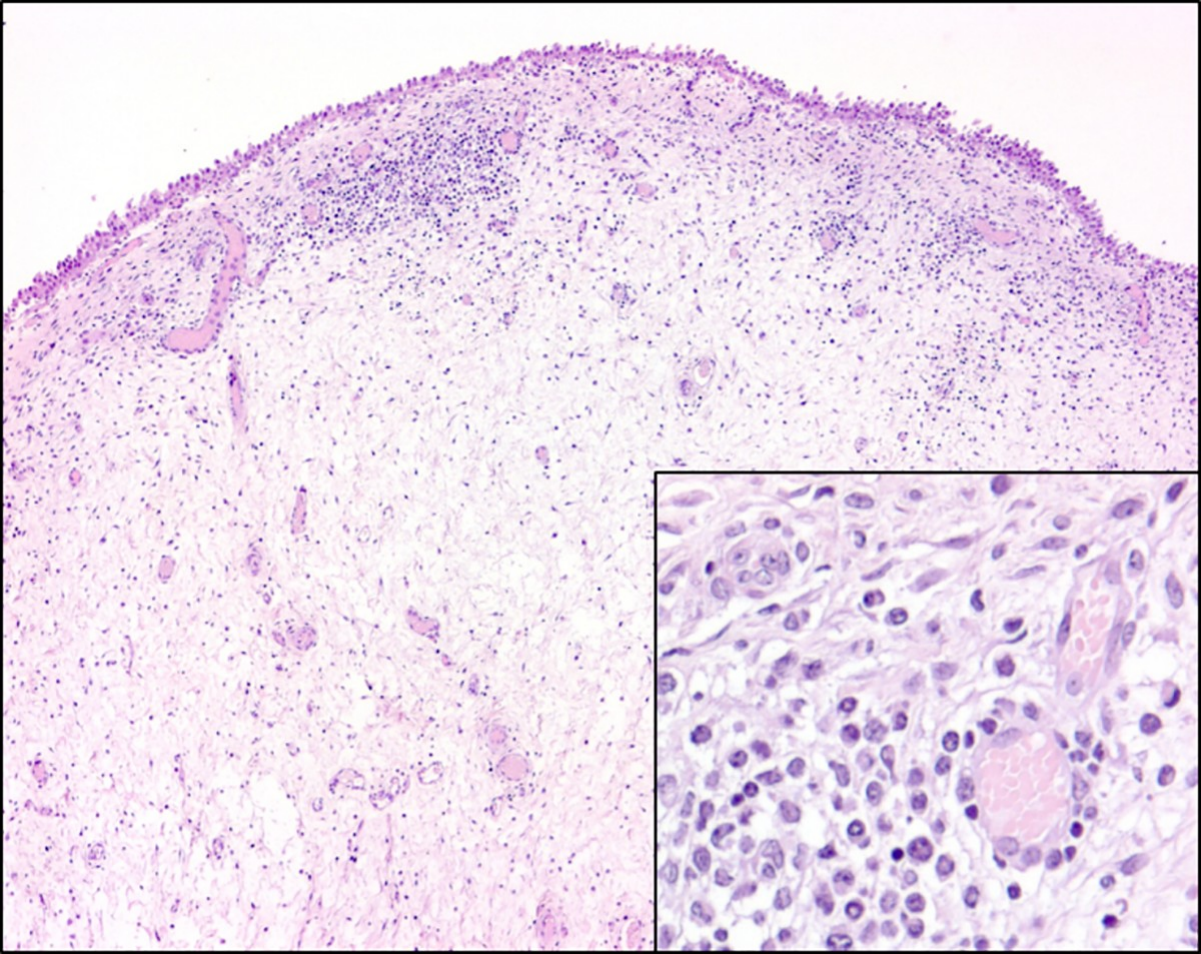
Urinary bladder mucosa of heifer 1567. The lamina propria is characterised by diffuse oedema and diffuse and follicular infiltration with mononuclear cells and neutrophils as well as multifocal proliferation of small blood vessels. Inset: Hypertrophic, cuboidal endothelial cells within proliferating capillaries in the lamina propria. Haematoxylin and eosin.

The urinary bladder samples of all four heifers were characterized by diffuse oedema in the lamina propria and submucosa (most prominent in heifers 1567 and 4849), capillary hyperplasia with hypertrophic cuboidal endothelium in the lamina propria (most prominent in heifers 1567, 4849, and 1550), and diffuse as well as follicular infiltration of the lamina propria with mononuclear cells (follicular cystitis, heifer 4848) (**Fig 13**). No histopathological changes were found in the remaining organs.

## Discussion

Intravenous administration of purified PTA revealed a modest elimination rate of PTA with a terminal half-life up to 6 hours and a MRT up to 4 hours. The Vss was approximately 1.3 L kg^-1^, indicating an almost equal distribution of PTA throughout the body. The profile was expected due to the amphiphilic properties of PTA, including a hydrophilic nucleus (glucose) and the lipophilic aglycone part (illudane skeleton) [39]. A rather large fraction of PTA was rapidly converted to non-toxic PTB. Usually, metabolites are formed at a rate that leads to an increase in plasma concentration followed by a decrease, when the formation is less than the elimination. In the actual case, PTB appeared at a peak concentration in the first blood sample 10 min after administration suggesting an immediate hydrolysis of PTA in the blood. Since PTA is rather unstable in aqueous solutions [24,25], it was speculated if a fraction of PTA might have been converted to PTB in the injection solution. To exclude this artefact, the intravenous studies were repeated under very strict conditions, where PTA was dissolved and injected intravenously within 2 minutes. The new studies demonstrated peak concentrations of PTA as well as PTB in the first blood sampling, and confirmed an immediate formation of PTB within the body after PTA administration.

The excretion of PTA and PTB in urine after intravenous administration was almost complete 8 hours after administration, representing 25–50% of the dose. The remaining parts of the dose may have transformed to other metabolites including ptaquilosin and dienone. PTA is very unstable under the alkaline conditions found in ruminants urine, and will degrade into PTB and other metabolites.

PTA was neither detected in plasma or urine after oral administration of bracken extract or dried bracken fern. PTB was observed in both single and repeated dose studies, indicating a degradation of PTA in rumen or a first pass metabolism. *In vitro* studies with rumen fluid confirmed that PTA is lost within few minutes in the rumen. Sterilized (boiled) ruminal fluid did not degrade PTA reflecting that enzymes and/or microorganisms are critical for PTA degradation. Besides PTB, other metabolites might have been formed as PTB only amounted for 10% of the added PTA 5 min after the start of incubation and 16% at the final sampling (120 min). PTA transformed into the dienone reacts as an electrophile with compounds such as amines, amino acids, nucleosides and nucleotides (Ojika et al. 1987). The transient peak of PTB observed in plasma in the one week repeated dose study after the morning dose (Fig 8) amounted for 10% of the PTA dose which correlated with the findings of the rumen incubation study. Thus, PTA is degraded very fast in rumen and only PTA metabolites are absorbed.

Fletcher *et al*. [20] measured PTA in plasma and tissues from calves fed *P*. *esculentum* (PTA dose of 4.5 μmol kg^-1^ b.w.) for 18 and 24 days and observed that the toxin accumulated within tissue. However, the authors did not measure PTA directly but transformed PTA to PTB (hydrolysis) followed by quantification of PTB. The dose of PTA ingested in the studies of Fletcher *et al*. [20] was almost identical to the single dose study presented in this paper. However, the peak concentration of PTB in plasma was 26 times higher suggesting the measured PTB concentration includes other PTA metabolites converted to PTB (e.g. ptaquilosin and the dienone). The daily dose used in the present study was also similar to that of Fletcher *et al*. in the repeated dose study, although PTB did not accumulate as shown by Fletcher and co-workers [20].

PTA has been found in milk samples 38 h after cows have been fed bracken [17,18]. In a study, one cow was fed 6 kg of freshly cut bracken daily for 7 days corresponding to a PTA dose of 540 μmol day^-1^ (≈ 1.1μmol kg^-1^), and PTA was found in milk at the concentration of 0.28 μM [17]. In another study, cows were fed bracken at higher doses of PTA (12, 23, 41 and 50 μmol kg^-1^) for 5 days [18]. At the dose of 23 μmol kg^-1^, the PTA excreted in milk achieved a plateau of 50 μM representing 9.6% of the dose. In both studies, PTA was determined indirectly by alkalizing the milk aqueous extracts to form PTB. However, in this procedure PTB might have originated from ptaquilosin and dienone intermediates rather than PTA directly or from PtB already present. Our studies indicate that PTA is not absorbed at all and, therefore, cannot be expected to be excreted in milk (Figs 7 and 8).

The pathological changes in liver and urinary bladder are well documented in cattle with enzootic haematuria [2,40,41]. All animals developed centrolobular hypertrophy of the hepatocytes, while 3 heifers were found to have multifocal portal fibrosis. In parallel, an increased AST activity was observed, while OCT and GD remained constant (Fig 10). It must however be noticed that a single animal exhibited increased GD levels. Change in plasma AST is associated with acute hepatitis and change in GD and OCT with hepatocellular necrosis [42]. In the study by Fletcher and co-workers mentioned above the plasma activity of AST, creatine kinase, GD and GGT were reported to be within reference values and no histological changes were found in the liver. The enzyme levels in cattle with chronic ingestion of bracken are also varying between studies [43,44]. The differences may be related to the different composition and concentration of other compounds present in bracken [20,21].

The presence of oedema and infiltration of mononuclear cells in the bladder tissue has been described previously in cattle with chronic ingestion of bracken [40,41]. Long-term exposure of bracken has been associated with chronic cystitis, which is one of the most frequent findings at slaughter in cattle grazing bracken [40,41]. The inflammation and proliferative changes in the urinary bladder mucosa can be associated with preneoplastic urinary bladder changes. These changes might be attributed to reactive PTA-metabolites as ptaquilosin and the dienone, PTA-like compounds such as caudatoside and ptesculentoside or other toxic compounds present in bracken [28,45].

## Conclusion

This is the first report of the toxicokinetics and urinary excretion of PTA after intravenous and oral administration. Furthermore, the related pathological changes in the liver and urinary bladder are described. PTA is rapidly converted to PTB and eliminated via urine when injected directly into the blood stream. Under natural conditions when cows are eating bracken, PTA is quickly hydrolysed in rumen to metabolites including PTB. A seven day exposure to PTA in a feeding trial with bracken was sufficient to induce liver lesions and pathological changes in the urinary bladder mucosa, which may be classified as pre-neoplastic. Reactive metabolites of PTA, other illudane glycosides or other unknown genotoxic substances may be the cause of these effects. Further studies with labelled compounds as well as detailed studies on the reactivity of PTA and its metabolites are required to give a full picture of the fate of PTA in cattle.

## Abbreviations

AUC: area under the curve
b.w.: body weight
C_max_: maximum concentration
LC-MS: high-performance liquid chromatograph coupled with a single quadrupole mass spectrometer
MTR: mean residence time
MTT: mean transit time
PTA: ptaquiloside
PTB: pterosin B
T_max_: time of maximum concentration
Vss: apparent volume of distribution
λz: terminal slope
AST: *aspartate* aminotransferase
BEH: bovine enzootic haematuria
GD: glutamate dehydrogenase
OCT: ornithine carbamoyltransferase (OCT).
